# Role of Cathepsin B Expression in Oral Squamous Cell Carcinoma: A Comprehensive Review

**DOI:** 10.7759/cureus.54267

**Published:** 2024-02-15

**Authors:** Shakti Sagar, Pravin Gadkari, K.M. Hiwale, Miheer M Jagtap, Suhit Naseri

**Affiliations:** 1 Pathology, Jawaharlal Nehru Medical College, Datta Meghe Institute of Higher Education & Research, Wardha, IND

**Keywords:** prognostic significance, therapeutic target, tumor invasion, cancer biomarker, cathepsin b, oral squamous cell carcinoma (oscc)

## Abstract

This comprehensive review delves into the intricate landscape of oral squamous cell carcinoma (OSCC) by examining the role of cathepsin B expression in its pathogenesis. OSCC, a prevalent and clinically significant oral malignancy, poses a considerable global health burden, necessitating a thorough exploration of its underlying molecular mechanisms. Cathepsin B, a lysosomal cysteine protease, emerges as a critical player in OSCC, influencing tumour initiation, invasion, and metastasis. The review begins with a brief overview of OSCC, emphasizing its epidemiological and clinical features, followed by exploring the significance of studying cathepsin B expression in this context. In the manuscript, the structure and function of cathepsin B are elucidated, providing a foundation for understanding its aberrant expression in OSCC. Clinical studies revealing correlations with tumour grade and stage, along with prognostic significance, are scrutinized, offering insights into the potential diagnostic and prognostic utility of cathepsin B. The biological functions of cathepsin B in OSCC, including its impact on tumour invasion and modulation of apoptosis, are comprehensively discussed. The *Therapeutic Implications* section explores targeting cathepsin B as a potential strategy, emphasizing the need for future research to overcome associated challenges. In the *Conclusion *section, the review synthesizes key findings, delineates implications for future research, and highlights the potential impact of cathepsin B on OSCC diagnosis and treatment, contributing to the ongoing efforts to advance our understanding of this complex malignancy, which is associated with a high mortality rate and improve clinical outcomes.

## Introduction and background

Oral squamous cell carcinoma (OSCC) primarily arises from the epithelial lining of the oral cavity, including the lips, tongue, gingiva, and palate. It ranks among the most common malignancies affecting the oral region, exhibiting diverse histopathological patterns and clinical behaviours. The etiological factors encompass a complex interplay of genetic predisposition, environmental exposures, and lifestyle choices, such as tobacco and alcohol consumption [1]. The dysregulation of various molecular pathways plays a pivotal role in the development and progression of OSCC. Cathepsin B, a lysosomal cysteine protease, has emerged as a potential key player in the intricate landscape of cancer biology. Understanding the significance of cathepsin B expression in OSCC is essential for unravelling its specific contributions to tumour initiation, invasion, and metastasis [2].

This comprehensive review aims to consolidate and analyse existing knowledge on the role of cathepsin B expression in OSCC. By synthesising findings from clinical studies, molecular investigations, and therapeutic implications, this review seeks to provide a comprehensive understanding of how cathepsin B influences the pathogenesis of OSCC. Moreover, the review aims to shed light on the potential diagnostic and therapeutic avenues that may arise from targeting cathepsin B in the context of OSCC management. Through this exploration, the ultimate goal is to contribute valuable insights that may inform future research directions and enhance the clinical management of OSCC.

## Review

Background

General Information on Oral Cancers

Oral cancer, encompassing the mouth and the posterior throat malignancies, manifests on the tongue, the mucosal lining of the mouth and gums, and various oral regions. It constitutes approximately three percent of all annually diagnosed cancers in the United States, with over 54,000 new cases reported in 2022 [3]. Predominantly affecting individuals over 40 years of age and displaying a higher incidence in men, risk factors for oral cancer encompass tobacco use, excessive alcohol consumption, human papillomavirus (HPV) infection, inadequate nutrition, and genetic predisposition. Clinical manifestations may include a white or red patch in the oral cavity, a non-healing lip or mouth sore, and bleeding, pain, or numbness in the lip or mouth [3,4]. Globally, oral cancer poses a significant health challenge, ranking among the 10th most prevalent cancer sites internationally. Cancers of the lip, oral cavity, hypopharynx, oropharynx, and larynx are known collectively as mouth and oral cancers. Of these, cancers of the lip and oral cavity are the most common, with more than 377,700 cases worldwide in 2020 [3]. It stands as the 16th most common malignancy and the 15th leading cause of death worldwide, with an estimated 378,500 new cases of intraoral cancer diagnosed annually across the globe. Unfortunately, regions with the highest prevalence of oral cancer often lack comprehensive descriptive information regarding its incidence, mortality, and prevalence [5].

Molecular and Cellular Basis of OSCC

OSCC development involves various genetic and molecular factors, reflecting a multistep process that significantly alters the normal functions of proto-oncogenes, oncogenes, and tumour suppressor genes [6]. One critical facet is the occurrence of genetic alterations, where multiple events contribute to the transformation of normal cells into cancerous ones. In addition, epigenetic modifications, such as DNA methylation and histone modifications, exert influence by inducing changes in gene expression, thereby contributing to the overall development of OSCC [7]. Molecular signaling networks play a pivotal role in the initiation and progression of OSCC. Essential genes and signaling pathways, such as the p16/Cyclin D1/pRb/p53 pathway, are instrumental in regulating cell cycle progression and suppressing tumour formation [7]. This emphasizes the intricate molecular mechanisms at play during carcinogenesis.

Human papillomavirus (HPV) can infect the mouth and throat and cause cancers of the oropharynx (back of the throat, including the base of the tongue and tonsils). This is called oropharyngeal cancer. HPV is thought to cause 70% of oropharyngeal cancers in the United States [7]. HPV is recognized as a critical driver in a subset of oropharyngeal squamous cell carcinomas, but its prevalence as a molecular driver in OSCC is not predominant [7]. However, understanding its role remains crucial for comprehensively understanding the diverse etiological factors contributing to OSCC. Furthermore, oral hygiene and microbiota significantly influence OSCC risk [8]. Poor oral hygiene and an imbalanced microbiota contribute to an increased susceptibility to OSCC, highlighting the importance of lifestyle and environmental factors in its development. As another contributing factor, genetic susceptibility underscores the role of individual genetic predisposition in OSCC development [8]. This combination of genetic alterations, epigenetic modifications, molecular signaling networks, HPV, oral hygiene, microbiota, and gene susceptibility collectively shapes the intricate landscape of OSCC pathogenesis. Recent advances in high-throughput genetic sequencing technologies underscore the evolving landscape of OSCC research. These technological breakthroughs provide a more comprehensive understanding of the key molecular signaling networks that underlie the initiation and progression of OSCC [7]. Recognizing these complexities at the molecular and cellular levels is imperative for developing targeted therapies and identifying biomarkers that can predict responses to treatment, ultimately paving the way for more effective management of OSCC.

Introduction to Cathepsin B and its Role in Cancer

Lysosomal cysteine proteinase cathepsin B plays a critical role in a number of human diseases and oncogenic processes, most notably cancer. The expression and trafficking patterns of cathepsin B undergo frequent alterations in cancer, with its plasma membrane and secreted forms believed to participate actively in invasion and metastasis [9]. Elevated levels of cathepsin B expression have been documented across diverse cancer types, including lung squamous cell carcinoma, prostate cancer, colorectal cancer, gliomas, melanomas, and breast cancer [10,11]. Recognized for its significance in tumorigenesis, angiogenesis, invasion, and metastasis, cathepsin B emerges as a crucial player in cancer progression [10]. Numerous clinical studies have substantiated the correlation between cathepsin B expression and disease progression, underscoring its potential as a prognostic indicator for various tumour types [9]. While targeting cathepsin B has demonstrated notable therapeutic benefits in cancer treatment, it is evident that singularly focusing on cathepsin B does not eliminate or suppress tumour growth, emphasizing the complexity of the underlying mechanisms [12,13].

Cathepsin B: structure and function

Structure of Cathepsin B

**Figure 1:**
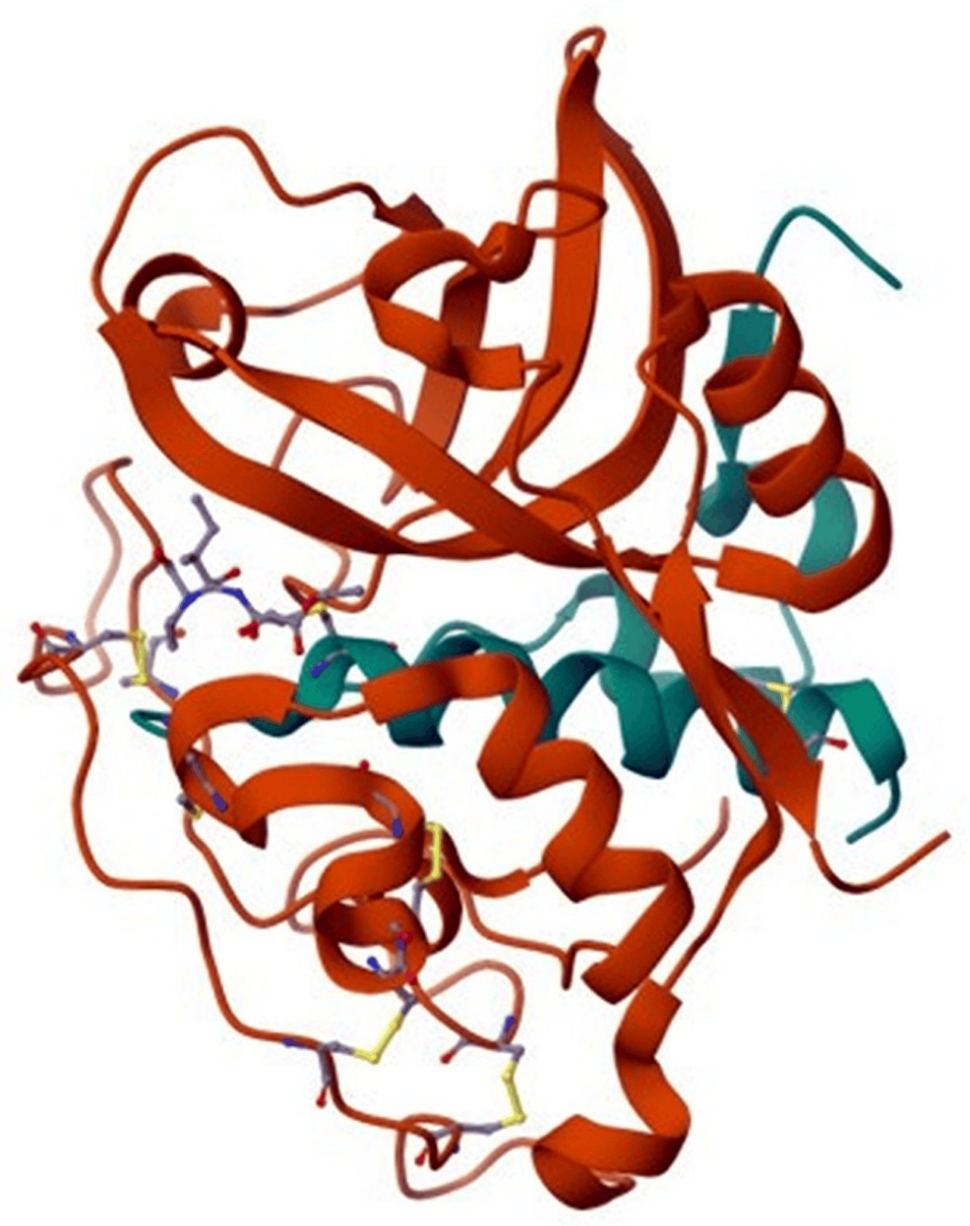
Crystal structure of cathepsin B inhibited This figure was taken from the open-source website [15].

Cathepsin B, classified as a cysteine protease, plays a pivotal role in intracellular proteolysis and is a member of the cysteine cathepsins family [11]. Initially synthesized as a pre-proenzyme comprising 339 amino acids, cathepsin B undergoes a series of processing steps to achieve its functional forms [14]. Following synthesis, the pre-proenzyme transforms into pro cathepsin B, a 43/46 kDa protein, during transit to the Golgi apparatus. Within this cellular compartment, the active form of cathepsin B is ultimately generated [14]. The mature cathepsin B form consists of a heavy chain (25-26 kDa) and a light chain (5 kDa) linked by a disulfide bond [14]. The enzyme's catalytic site, crucial for its activity, is located at the interface between the two lobes, featuring a catalytic triad comprising cysteine, histidine, and aspartic acid. This triad facilitates enzymatic activity, with cysteine interacting with histidine to catalyze peptide bond cleavage [11]. An indispensable structural element of cathepsin B is the 18-residue insertion, the occluding loop, which significantly contributes to substrate binding and enzyme activity [14]. Cathepsin B participates in various physiological and pathological processes, including intracellular proteolysis, the potentiation of other proteases, cell autolysis, and tissue self-digestion [11]. Elevated levels of cathepsin B are notably associated with poor prognosis and increased aggressiveness in various cancers, positioning it as a potential biomarker for cancer diagnosis and prognosis [11]. Figure 1 illustrates the crystal structure of inhibited cathepsin B, visually representing its structural characteristics.

Physiological Functions of Cathepsin B

Cathepsin B, a lysosomal cysteine protease, exhibits diverse functionalities across various physiological processes crucial for cellular and tissue homeostasis. One prominent role involves its participation in the proteolytic processing of prohormones and proenzymes, exemplified by its involvement in the cleavage of thyroglobulin (Tg). This enzymatic activity leads to the generation of essential thyroid hormones thyroxine (T4) and triiodothyronine (T3), thus playing a vital role in regulating thyroid function and hormonal balance within the body [14]. Furthermore, cathepsin B assumes a central position in the intricate process of antigen processing, a fundamental mechanism for the immune system. Cathepsin B generates peptides that can be presented to immune cells by breaking down antigens. This facilitation of antigen presentation is essential for mounting an effective immune response against foreign substances, thus enhancing the body's defense mechanisms [14].

The enzyme also plays a significant role in inflammatory responses against antigens, actively contributing to eliminating pathogens. In doing so, cathepsin B aids in maintaining immune homeostasis, highlighting its importance in orchestrating immune defences against external threats [14]. In addition to its immune-related functions, cathepsin B is involved in tissue remodelling. This dynamic process, crucial for maintaining the integrity and functionality of various tissues, relies on cathepsin B's ability to participate in the degradation of extracellular matrix components. Through its role in tissue turnover and renewal, cathepsin B contributes to the dynamic equilibrium of tissue structures [14]. Moreover, cathepsin B is intricately tied to apoptosis, a programmed cell death process crucial for maintaining tissue homeostasis. By eliminating damaged or abnormal cells, cathepsin B contributes to regulating cellular populations, preventing the accumulation of potentially harmful cells within the body [14]. Lastly, cathepsin B's involvement in lysosomal exocytosis underscores its role in cellular waste management and recycling of cellular materials. With carboxy dipeptidyl activity within lysosomes, cathepsin B facilitates protein degradation and autophagy, which is essential to maintaining cellular homeostasis [16].

Aberrant Expression in Cancer

N-cadherin: The aberrant expression of N-cadherin has been identified in numerous cancers, including lung cancer, breast cancer, prostate cancer, and squamous cell carcinoma [17]. While N-cadherin typically plays a crucial role in various tissues' developmental and functional regulation, its abnormal expression is closely linked to the critical aspects of cancer progression. This includes its involvement in cellular transformation, adhesion, apoptosis, angiogenesis, invasion, and metastasis. The association of N-cadherin with these critical functions suggests its potential as a therapeutic target for impeding tumour invasion and metastasis [17].

Paxillin: Abnormal expression of paxillin has been noted across a spectrum of human malignancies, including melanoma, breast cancers, gastric cancers, and colorectal cancers [18]. Paxillin plays a regulatory role in diverse biological functions, including tumour migration, heterotypic adhesion, invasion, survival, and angiogenesis, contributing to the malignant development of tumours through various molecular mechanisms [18]. The correlation between aberrant paxillin expression in cancer patients and unfavourable clinical outcomes, such as poor prognosis, tumour occurrence, and metastasis, further underscores its significance as a potential prognostic marker and therapeutic target [18].

Cathepsin B: In the context of OSCC, there is an observed increase in cathepsin B expression, and OSCC patients exhibiting cathepsin B expression tend to experience shorter overall survival compared to those without such expression [19]. Cathepsin B, a cysteine protease, plays a crucial role in intracellular proteolysis and is overexpressed in several human cancers, indicating its potential involvement in tumorigenesis [19]. This highlights cathepsin B as a potential biomarker for OSCC prognosis and a candidate for targeted therapeutic interventions.

Reg protein: Aberrant expression of the Reg protein has been identified in various human diseases, including cancer and inflammation [20]. The Reg gene, acting as a potential oncogene, demonstrates abnormal expression linked to various cancer-related processes. The involvement of the Reg protein in diseases suggests its potential role as a diagnostic marker and therapeutic target in cancer and inflammatory conditions [20]. Understanding the molecular mechanisms underlying Reg protein dysregulation could pave the way for targeted interventions in managing associated diseases.

Expression of cathepsin B in OSCC

Evidence from Clinical Studies

Overview of relevant studies: Numerous investigations have explored the expression of cathepsin B in OSCC and its potential implications for the disease. A 2022 study aimed to assess cathepsin B salivary levels across different OSCC patients' histological grades. The study revealed an elevated expression of cathepsin B in various histological grades of OSCC, indicating a potential association with the malignancy [21]. Another study from 2016, published in the National Center for Biotechnology Information, discovered that OSCC patients exhibiting cathepsin B expression had shorter overall survival, hinting at a plausible correlation between cathepsin B expression and clinical outcomes in OSCC patients [19]. A figure from a study depicted a Kaplan-Meier survival curve, illustrating the connection between cytoplasmic cathepsin B expression in primary tumours and the survival of 280 OSCC patients. While these studies propose cathepsin B as a potential biomarker for predicting cancer progression in OSCC, they acknowledge limitations, such as small sample sizes and the absence of patients with benign lesions or other chronic inflammatory oral diseases [19]. Future investigations with larger sample sizes and more diverse patient populations are imperative to further validate cathepsin B's role in OSCC.

Correlation with tumour grade and stage: Cathepsin B expression has been identified as correlated with tumour grade and overall survival in OSCC. A 2016 study on demonstrated that heightened cathepsin B expression correlates with higher tumour grades and significantly poorer overall survival in 280 OSCC patients [19]. This suggests the potential utility of cathepsin B expression as a prognostic indicator in OSCC. In addition, the observed increased cathepsin B expression in varying histological grades of OSCC further supports its association with cancer progression [22]. These findings underscore the potential significance of cathepsin B as a biomarker for predicting tumour grade and clinical outcomes in OSCC.

Prognostic significance: Cathepsin B expression in OSCC has been linked to prognostic significance. A 2016 study found that increased cytoplasmic cathepsin B expression in primary tumours was correlated with significantly poorer overall survival in 280 OSCC patients [19]. In addition, the same study noted that buccal mucosa squamous cell carcinoma patients with positive cathepsin B staining experienced significantly lower overall survival [19]. However, a 2016 study in the Journal of Biological Chemistry, focusing on colorectal tumour progression, demonstrated that elevations in cathepsin B expression were associated with tumour malignancy [23]. Furthermore, a 1994 immunohistochemical study in Cancer found that higher-grade expression of cathepsin B was significantly linked to shorter survival in nonsmall cell lung cancer [24]. These findings suggest that cathepsin B expression may hold prognostic significance in various cancer types, including OSCC.

Molecular Mechanisms Regulating Cathepsin B Expression in OSCC

Transcriptional regulation: Transcriptional regulation governs the control of gene expression at the transcription level, encompassing the synthesis of RNA from DNA. In the context of OSCC, the molecular mechanisms orchestrating cathepsin B (CTSB) expression involve activating specific transcription factors binding to the promoter region of the CTSB gene, thereby modulating its expression. Studies have identified the transcription factor Sp1 as a critical player in regulating CTSB expression in OSCC [19]. Moreover, research indicates that activation of the PI3K/Akt signalling pathway can enhance CTSB expression in OSCC [22]. In addition, the transcription factor E2F1, known for its regulatory role in CTSB expression in other cancer types, emerges as a potential participant in OSCC [25]. These findings underscore the complexity of CTSB transcriptional regulation in OSCC, involving the activation of diverse signalling pathways and transcription factors.

Post-translational modifications: The expression and activity of cathepsins, including cathepsin B, undergo intricate regulation through various post-translational modifications. These modifications encompass alterations in pH, maturation processes, cellular localization, and interactions with other molecules. The enzymatic activity of cathepsins, pivotal in processes, such as inflammation, tumour progression, and metastasis, is notably influenced by post-translational modifications [26]. Furthermore, the post-translational processing and maturation of cathepsins involve nuanced steps, including the removal of pre-peptides, translocation to distinct cellular compartments, and glycosylation of specific amino acid residues [27]. It has been proposed that cathepsin B undergoes post-translational modifications after its synthesis, exerting an impact on its function and activity [22]. These revelations underscore the critical role of post-translational modifications in governing the expression and function of cathepsin B, not only in pathological conditions but also in various physiological processes.

Biological functions of cathepsin B in OSCC

Promotion of Tumour Invasion and Metastasis

Cathepsin B is crucial in promoting tumour invasion and metastasis across a spectrum of cancers, including hepatocellular carcinoma (HCC), breast cancer, melanoma, lung cancer, and head and neck cutaneous squamous cell carcinoma (SCC). Research reveals the upregulation of cathepsin B in HCC tissues, where it is released by tumour cells, thereby promoting migration and invasion within the context of HCC [28]. In breast cancer, melanoma, and lung cancer, heightened cathepsin B activity has been correlated with increased tumour invasion and metastasis [29]. Similarly, in head and neck cutaneous SCC, the overexpression of cathepsin B has been associated with cancer invasion and metastasis [30]. In addition, cathepsin B has been identified as a promoter of the spread and invasion of OSCC [31]. These collective findings underscore the pivotal role of cathepsin B in facilitating tumour invasion and metastasis across various cancer types.

Modulation of Apoptosis and Cell Survival

Numerous studies have delved into the intricate modulation of apoptosis and cell survival within OSCC. Notably, Hispolon, a compound derived from specific fungi, has demonstrated its capability to induce apoptosis in OSCC cells through the JNK/HO-1 pathway and caspase-dependent mechanisms. This underscores its potential as an anticancer agent with promising implications for OSCC treatment [32]. In addition, investigations have shed light on the influence of various genes and signalling pathways, including BCLAF1 and the Fas-mediated apoptosis pathway, in shaping the apoptotic responses of OSCC cells [33,34]. In a significant stride towards novel therapeutic approaches, a study showcased the ability of nano emodin transfersome to augment Fas-mediated apoptosis in head and neck squamous cell carcinoma (HNSCC) cell lines, introducing the possibility of modulating apoptosis through innovative delivery systems [35]. These collective findings contribute substantially to an enhanced comprehension of the underlying mechanisms involved in the modulation of apoptosis and cell survival in oral cancer. This knowledge holds significant promise for developing targeted therapeutic strategies to address the complexities of OSCC.

Interaction with the Tumour Microenvironment (TME)

Cathepsin B engages with the intricate dynamics of the tumour microenvironment (TME) through multiple avenues. Tumour cells strategically upregulate cysteine cathepsins, including cathepsin B, to bolster their survival, proliferation, motility, and invasive capabilities. Once released into the extracellular space, these cathepsins actively remodel the extracellular matrix (ECM) and break down specific chemokines. Concurrently, tumour cells orchestrate the production of cytokines, steering myeloid cells towards an immunosuppressive phenotype, thereby fostering heightened synthesis of cysteine cathepsins in tumour-associated macrophages (TAMs) and myeloid-derived suppressor cells (MDSCs). The role of immune cell-supplied cathepsins in fueling tumour progression has been extensively explored, with monocytic cells notably elevating cathepsin expression upon interaction with the TME [36]. Moreover, cathepsin B's involvement in tumour-stromal interactions is apparent, as these interactions induce the expression of cathepsin B. The TME also witnesses the release of cathepsins, which may interact with pericytes, versatile mural cells within the TME [37,38]. Within the context of OSCC, investigations have unveiled a correlation between the expression of cathepsin B and cancerization in OSCC, both in vitro and in vivo. This observation underscores the potential role of cathepsin B in influencing the progression of the disease [25]. Cathepsin B assumes a multifaceted role within the TME, influencing diverse facets of tumour progression and interactions with the surrounding microenvironment.

Therapeutic implications

Targeting Cathepsin B as a Therapeutic Strategy

Inhibition of cathepsin B activity: Inhibition of cathepsin B activity has been a research focus due to its potential role in cancer therapy. Several protein inhibitors of cathepsin B have been described, some of which are of endogenous origin and function as regulators of cathepsin B activity in the cell [39]. Exogenous protein inhibitors of cathepsin B have also been isolated from various natural sources [39]. These inhibitors can be classified into reversible and irreversible types, with different mechanisms of action [39]. One example of a potent and selective inhibitor of neutral pH 7.2 cathepsin B activity is Z-Arg-Lys-AOMK [40]. This inhibitor displays high specificity for cathepsin B and is cell permeable, inhibiting intracellular cathepsin B [40]. Another study developed a selective neutral pH inhibitor of cathepsin B based on its pH-dependent cleavage properties [40]. This irreversible inhibitor displays nanomolar potency with 100-fold selectivity for inhibition of cathepsin B at pH 7.2 compared to pH 4.6 [5]. In cancer patients, elevated cathepsin B activity correlates to poor therapy outcomes, and the use of cathepsin B inhibitors has been found to reduce both tumour cell motility and tumour growth [39]. However, targeting cathepsin B alone may not be sufficient to eliminate tumours, and a multifaceted approach targeting multiple molecules or using a combination of chemotherapy or radiation along with targeting cathepsin B may be more appropriate [41]. Inhibition of cathepsin B activity has potential therapeutic implications in cancer treatment. Several protein inhibitors of cathepsin B have been developed, and research is ongoing to improve their specificity and potency. Combining cathepsin B inhibitors with other cancer therapies may enhance their efficacy in treating cancer.

Development of cathepsin B inhibitors: The development of cathepsin B inhibitors is an active area of research due to their potential as a therapeutic target for cancer and other diseases. Several studies have reported the development of small-molecule inhibitors of cathepsin B, including CA074, E6446, and CA045 [42]. However, none of these inhibitors have shown strong enough anticancer effects to be clinically available, and there are currently no FDA-approved cathepsin B inhibitors for cancer treatment [42]. Developing potent and selective low-molecular cathepsin B inhibitors relies on the detailed expertise of preferred amino acid and inhibitor residues [43]. A study published in the National Center for Biotechnology Information 2001 described the structure-based design of a cathepsin B-specific inhibitor [44]. Another study published in 2016 found that limiting the inhibitor effect to the extracellular space or preventing lysosomal exocytosis may be a better cathepsin B targeting strategy [41]. In addition, a recent study published in 2023 developed a peptide-based nano-sized cathepsin B inhibitor with a remarkable therapeutic potential and low toxicity [42]. These findings suggest that the development of cathepsin B inhibitors is a promising area of research for treating cancer and other diseases.

Challenges and Future Directions in Cathepsin B-Targeted Therapy

Targeting cathepsin B as a therapeutic strategy for cancer treatment faces several challenges and requires further research. One of the significant challenges is the complexity of cysteine cathepsins' immunological processes, making it difficult to achieve selectivity and avoid off-target effects [45]. Another challenge is the specificity of the compound, as cathepsin B inhibitors may also inhibit other enzymes [41]. Moreover, targeting cathepsin B alone may not be sufficient to eliminate tumours, and a multifaceted approach targeting multiple molecules or using a combination of chemotherapy or radiation along with targeting cathepsin B may be more appropriate [12]. However, recent advances in imaging tools that target cathepsin B have been proposed, which can help distinguish tumour boundaries and guide surgical early diagnosis of tumours [46]. In addition, pro-drugs and other target therapeutic agents that respond to cathepsin B have been proposed and used in cancer treatment, and some of them are already on the market [46,47]. Therefore, further research is needed to develop more selective and potent inhibitors for cathepsin B and to investigate the clinical utility or benefit of any cathepsin B inhibitors [41].

## Conclusions

This comprehensive review of cathepsin B expression in OSCC reveals critical insights that significantly contribute to our understanding of this malignancy. The summarized key findings emphasize cathepsin B's association with tumour grade and stage, shedding light on its multifaceted roles in OSCC pathogenesis. As a potential biomarker, cathepsin B holds promise for predicting disease outcomes and guiding therapeutic decisions. At the same time, its involvement in promoting invasion and modulating apoptosis underscores its significance in OSCC progression. Future research should delve deeper into the intricate molecular mechanisms and signalling pathways regulated by cathepsin B in OSCC, paving the way for innovative treatment strategies. The identified implications for future research underscore the need for continued exploration of cathepsin B and its interactions within the tumour microenvironment. Lastly, the potential impact on OSCC diagnosis and treatment is substantial, suggesting that integrating cathepsin B assessment into diagnostic protocols may enhance precision, offering personalized treatment approaches for improved clinical outcomes in individuals with OSCC.
